# Dietary duration and composition differentially influence mitochondrial activity and gene expression in a tissue-specific manner in aged rats

**DOI:** 10.1016/j.tma.2025.11.001

**Published:** 2025-11-28

**Authors:** Brea J. Ford, Anisha Banerjee, Sarah Ding, Anna A. Caton, Ashley Grothaus, Sara N. Burke, Abbi R. Hernandez

**Affiliations:** a University of Alabama at Birmingham, Heersink School of Medicine, Department of Medicine, Division of Gerontology, Geriatrics and Palliative Care, Birmingham, AL, USA; b University of Florida, Department of Neuroscience, Gainesville, FL, USA

**Keywords:** Fasting, Ketogenic diet, Hippocampus, Muscle, Liver

## Abstract

Mitochondrial dysfunction is a hallmark of aging, affecting multiple systems and tissues, contributing to impairments in function. The resultant decreases in energy availability, along with increased oxidative stress, may be attenuated through diet. Fasting paradigms (including time restricted feeding (TRF)) and ketogenic diets (keto) both influence mitochondrial function, potentially mitigating these effects. However, the duration and modality of dietary intervention required for ameliorating age-related mitochondrial impairments remain unknown. Therefore, this study investigated the effects of a chronically (8–24 months; cTRFc) and acutely (22–24 months; aTRFc) administered TRF diet with standard macronutrients, as well as a chronically (8–24 months) administered TRF with ketogenic macronutrients (cTRFk), on mitochondrial activity and gene expression in aged male rats across tissues (brain, liver, muscle). Despite some synergy across the chronic diet groups, keto and TRF duration influenced mitochondrial function in a tissue- and diet-specific manner. Mitochondrial complex II activity was higher in cTRFk rats within the liver. Mitochondrial complex IV activity was lower in muscle and hippocampal tissue in both chronic TRF-fed groups. Relatedly, expression of the complex IV-related gene *Cox2* increased within the CA3 subregion of the hippocampus of cTRFk. In this same region, expression of the mitochondrial biogenesis related gene *Pgc1a* was increased in cTRFc diet rats only. Within the liver, *Cox5b* expression increased in both groups of chronic TRF rats. Together, these findings highlight complex, tissue-specific responses to long-term dietary interventions, emphasizing the need for further research to develop targeted nutritional strategies for enhancing mitochondrial function and metabolic health in aging populations.

## 1. Introduction

Mitochondria, dynamic and multifaceted organelles responsible for cellular energy production, undergo a series of changes and functional alterations as organisms age. The mitochondrial theory of aging posits that cumulative damage to mitochondrial DNA and functional decline in mitochondria contribute to cellular aging by compromising energy production and increasing oxidative stress [1,2]. These age-related changes impair cellular function across many organ systems, including the central nervous system (CNS) [3], skeletal muscle [4] and liver [5]. In fact, mitochondrial dysfunction is a key hallmark of advanced aging [6] and may directly impact many age-related disease states [7]. For example, mitochondrial dysfunction is a key factor in sarcopenia, the age-related decline in muscle mass and function [8] and mitochondrial-related gene expression is significantly altered within the aged liver [9]. Within the brain, impairments in mitochondrial function in aging result from decreased electron transfer rates in complexes I and IV, among other factors [10].

Many dietary interventions, including ketogenic diets (keto) and time restricted feeding (TRF)/intermittent fasting (IF) have beneficial effects on mitochondrial function [11–13]. These dietary regimens, especially keto diets, induce a metabolic switch from glucose to fatty acid and ketone body utilization, which may optimize mitochondrial function and energy production. Fasting activates pathways that enhance mitochondrial quality control, such as autophagy and mitophagy, which help remove damaged mitochondria and promote the generation of new, healthy mitochondria [14,15]. Additionally, maximal citrate synthase activity within heart, liver and brain tissue is higher on a keto diet relative to not only control-fed rats, but to calorically restricted and intermittently fasted rats as well [16]. Even in the absence of reduced caloric intake (as implemented herein), lifelong TRF improves morbidity and mortality in mice [17].

As dietary programs for the treatment of various health conditions, including the attenuation of age-related impairment, gain popularity, it is imperative that longer term implications of these diets are investigated. Therefore, in this study we used tissue from rats fed in three different paradigms from young adulthood (~8 months; post puberty) to advanced age (~24 months): Two groups were fed a Chronic TRF diet, one with control macronutrients (Chronic Control) and one with ketogenic macronutrients (Chronic Keto). The third group was control-fed rats on *ad libitum* feeding until 22 months of age, at which point they were placed on an acute (~2–3 month) TRF paradigm (Acute Control). Brain, liver and muscle tissue were collected for the assessment of mitochondrial function. Because mitochondrial dysfunction is particularly evident within hippocampus, analyses focused on this brain region [18–21].

Our findings highlight the complex, tissue-specific responses to long-term dietary interventions in aged male rats. As dietary strategies for improving health gain popularity, studies investigating extremely long-term use of these methods are imperative to understand potential health outcomes and to develop personalized nutritional strategies targeting specific aspects of impaired function and disease.

## 2. Materials and methods

### 2.1. Subjects, diet and tissue collection

The data presented herein used tissues from subjects whose behavioral status, as well as weight gain and other physiological parameters, has been previously published [22]. Male Fisher 344 x Brown Norway F1 (FBN) Hybrid rats (n = 31) were obtained from the National Institute on Aging colony at Charles River. Beginning at 8 months of age, rats were housed individually on a reverse 12-h light cycle with *ad libitum* access to water. Rats were divided into 3 dietary groups: 1) Acute TRF (fed with unrestricted access to standard (control) rodent chow through 21 months of age and then fed TRF until sacrifice), 2) chronic TRF rats that were fed 51 kcal/day of control diet chow (Chronic Control; 16 % fat, 65 % carbohydrates, and 19 % protein; Lab Supply 1810727) from 8 months old onward and 3) chronic TRF rats that were fed 51 kcal/day of a ketogenic diet (Chronic Keto; 76 % fat, 4 % carbohydrates, and 20 % protein; Neobee 895) from 8 months onward. Whether acute or chronic, the TRF regimen utilized herein was once daily meal feeding (at approximately 15:00) during rats’ dark cycle. We’ve previously observed that rats fed with this paradigm consume their entire meal within a 3-h window. However, as previously reported in Ref. [22], this amount of calories per day is sufficient to continue to promote weight gain across the lifespan in these subjects. Thus, this paradigm allows for the separation of effects of chronic caloric restriction from chronic TRF, as in Ref. [17]. However, at 21 months of age, all rats were modestly food restricted (~15 %) with the TRF protocol to encourage participation in a behavioral task for approximately 2 months, the results of which have been previously published [22]. Tissue was collected from all rats, regardless of diet group, at approximately 24 months of age.

At the time of sacrifice, rats were rapidly sedated with isoflurane and euthanized by rapid decapitation. The liver and the tibialis anterior muscle were isolated, rinsed in cold PBS and frozen on dry ice. Brains were rapidly extracted and immediately frozen on dry ice. All tissue was stored at −80 ° C until use. To micro dissect the different hippocampal (HPC) subregions, brains were sliced at approximately 300 μm thickness in a cryostat and a 1-mm tissue biopsy punch tool was utilized to collect CA1, CA3 and DG in separate tubes. Several punches from each region were collected and pooled. All samples from liver, muscle and HPC utilized for PCR were from the same subjects. Moreover, liver and muscle tissue from the same subjects were utilized for the mitochondrial complex activity data. However, because tissue was collected throughout 2021, severe COVID lockdowns prevented the collection of all tissues from all subjects (see Table 1 below for group counts), and HPC samples were not collected from the same cohort of rats. Samples used for HPC mitochondrial kinetics were collected from a separate cohort of rats treated the exact same way by the same lab group.

### 2.2. Mitochondrial activity assays

Mitochondrial complex activity assays were performed by the UAB Mitochondrial Core as previously published [23,24]. Briefly, Complex I activity was analyzed on a DU800 spectrophotometer using 2,6-dichlorindophenol (DCIP) as the terminal electron acceptor at 600 nm with the oxidation of NADH reducing artificial substrates Coenzyme Q10 that then reduces DCIP. Complex II activity was analyzed as the reduction of DCIP at 600 nm with succinate as the substate. Complex III activity was assessed in the mitochondria by measuring the rate of conversion of cytochrome *c* from its oxidized form to reduced form with ubiquinone as the substrate. Complex IV activity was measured by the oxidation of cytochrome *c* at 550 nm. Although mitochondrial citrate synthase levels did not differ between any groups within any tissues (data not shown), all data were normalized to mitochondrial citrate synthase levels to account for any variations in mitochondrial content between samples.

### 2.3. PCR

Due to previous work demonstrating gene expression alterations following keto in aged rats are subregion dependent [25], HPC tissues were micro-dissected into dentate gyrus (DG), CA1 and CA3 subregions prior to analysis. 30 mg of tissue was treated with 500ul of cold Trizol and allowed to incubate at room temperature for 10 min to allow complete dissociation of nucleoproteins. Tissue was further treated with 2-mercaptoethanol, vortexed and further incubated to allow for phase separation. The top layer (containing RNA) was then further purified with multiple cycles of ethanol washes before concentration determination via Nanodrop/One Viewer software. Superscript VILO cDNA synthesis kit (catalog 11754050) was utilized for cDNA generation. Samples were stored at −20 °C until use. Genes, listed in Table 2, were selected based on the results of the mitochondrial complex activity assays. In addition to subunits from complexes II and IV, markers of mitochondrial biogenesis were also utilized [26].

Gene expression was determined using the QuantStudio 6 Pro PCR system. PCR amplification reaction procedure consisted of the following steps: initial denaturation at 50 °C for 2 min followed by denaturation at 95 °C for 10 min, followed by 40 cycles of denaturation at 95 °C for 15s and extension at 60 °C for 60s. Relative gene expression for each sample was determined via QuantStudio Design and Analysis (DA)2.8 software. Samples not passing DA quality control were excluded. Actin beta (*Actb*) expression was used as a housekeeping gene for each subject and triplicate values were averaged per subject. All data were transformed to percent expression relative to the Acute Control group.

### 2.4. Statistical analysis

All data presented herein are expressed as group means ± SEM. Quantification of complex activity and relative gene expression was analyzed for each tissue separately, as expression greatly varies across tissue type. Multifactorial ANOVAs were utilized for each measure investigating effects of diet group as a fixed factor (across the 3 groups: Acute Control, Chronic Control and Chronic Keto). For complex activity data, the parent ANOVA included normalized complex I, II, III, IV and IV activity as dependent variables. For PCR data, the parent ANOVA included each gene (*Sdha, Pgc1a, Tfam, Cox1, Cox2, Cox5b*) as dependent variables. Post-hoc analyses for each assay were run via one-way ANOVAs, using Tukey’s correction for multiple comparisons. Null hypotheses were rejected at the level of p ≥ 0.05 and all analyses were performed with the Statistical Package for the Social Sciences v30 (IBM, Armonk, NY) or GraphPad Prism version 10.4.1 for Windows (GraphPad Software, La Jolla, California USA).

## 3. Results

### 3.1. Mitochondrial activity of complexes II and IV were differentially affected by dietary intervention across tissues

Mitochondrial complex activity was quantified for complexes I-V and all data were normalized to mitochondrial citrate synthase activity (despite no group differences in this across all tissues, p = 0.38; data not shown). Main effects of dietary group (Acute Control, Chronic Control and Chronic Keto) were investigated via multi-variate ANOVA (dependent variables = 5 complexes, fixed factor = diet grouping).

**Fig. 1.**
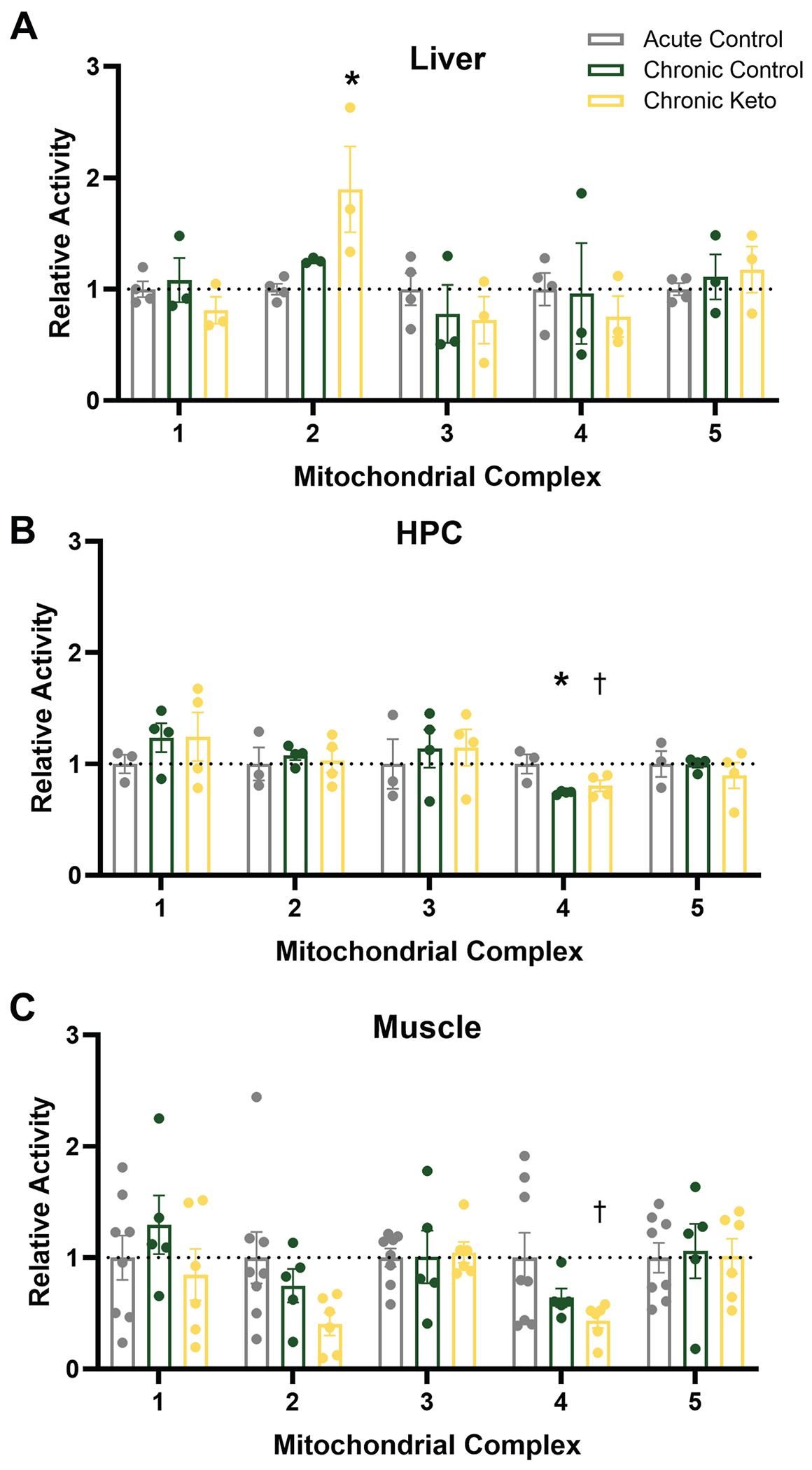
Mitochondrial complex I-IV activity across liver, hippocampus (HPC), and muscle tissues. Relative activity of mitochondrial complexes I-IV in Acute Control, Chronic Control and Chronic Keto in (A) liver, (B) HPC and (C) muscle (data normalized to Acute Control). All values represent group means ± SEM, *indicates a significant difference (p < 0.05) from Acute Control group, † indicates a trend for a difference (p ≤ 0.07) from Acute Control group.

There was a main effect of diet group on complex II activity within the liver (F_(2, 7)_ = 5.4, p = 0.04; Fig. 1A). Post-hoc analyses indicate Chronic Keto complex II activity was significantly higher than Acute Control (p = 0.03), but Chronic Control activity did not significantly differ from Acute Control, despite a strong trend (p = 0.064), nor did Chronic Control activity differ from Chronic Keto (p = 0.15). Together, these results demonstrate a stronger effect of keto diets than TRF on complex II activity in the liver.

There was a significant main effect of diet group on complex IV activity only within the HPC (F_(2, 10)_ = 6.37, p = 0.02; Fig. 1B). Within the HPC, post-hoc analyses revealed complex IV activity as significantly lower in the Chronic Control group relative to Acute Control (p = 0.02) and strongly trended towards the same in Chronic Keto relative to Acute Control (p < 0.07). The two Chronic TRF groups did not differ from one another (p = 0.66), indicating the main effect on complex IV activity within the HPC was driven by TRF duration, not macronutrient group.

Lastly, there was also a strong trend for an effect of diet within muscle tissue (F_(2, 18)_ = 3.00, p = 0.08; Fig. 1C), though post-hoc analyses did not reveal significant differences between any of the 3 groups. Complex activity did not differ in any of the other complexes investigated (I, III and V) by diet (p > 0.29 for all comparisons).

### 3.2. Mitochondrial-related gene expression was differentially affected by dietary intervention across tissues

**Fig. 2.**
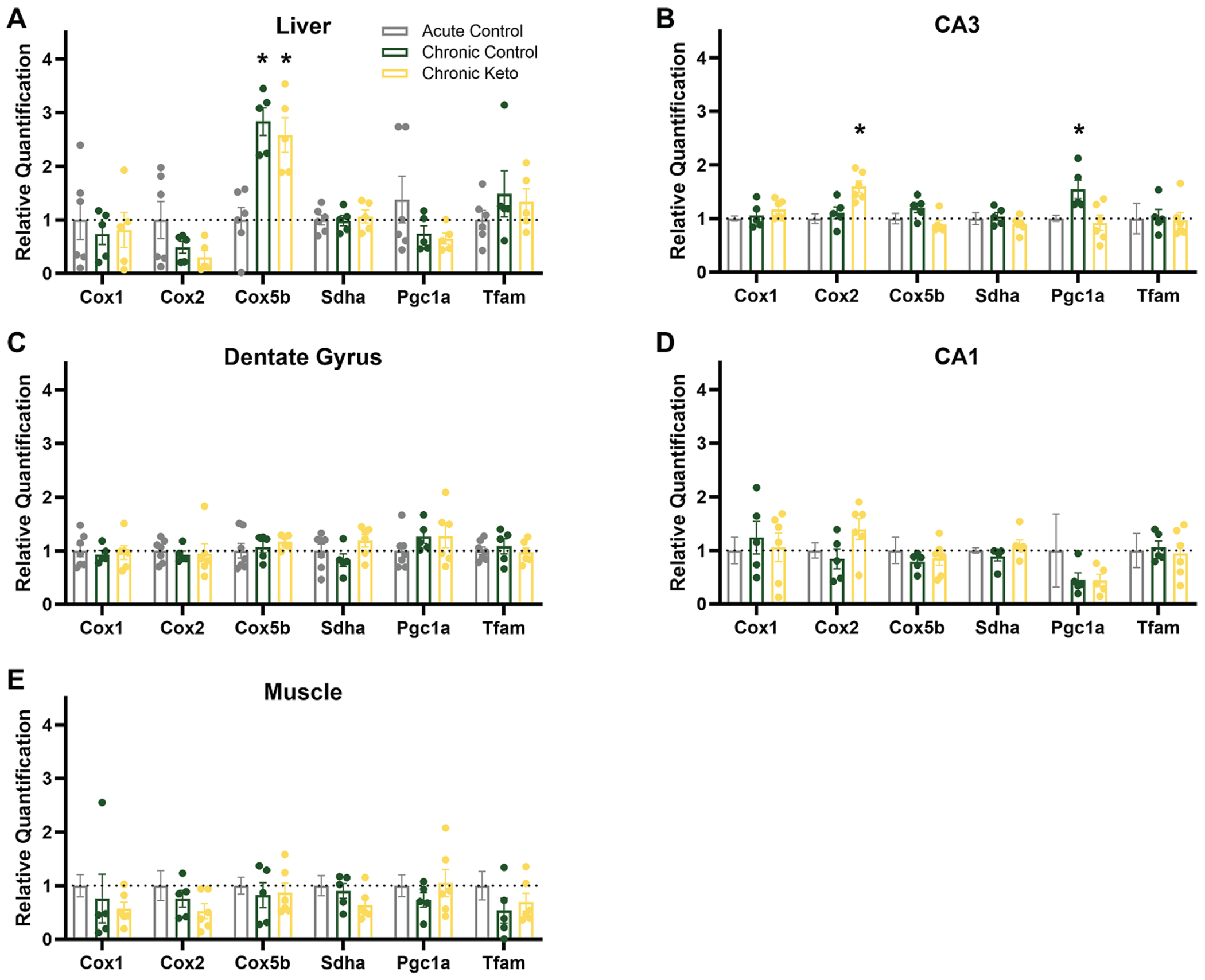
Expression of mitochondrial-related genes across tissues. Relative quantification of gene expression in Acute Control, Chronic Control and Chronic Keto in (A) liver, (B) CA3, (C) CA1, (D) dentate gyrus, and (E) muscle, normalized to Acute Control. All values represent group means ± SEM, *indicates a significant difference (p < 0.05) from Acute Control group, † indicates a strong trend for a difference (p ≤ 0.07) from Acute Control group.

Main effects of dietary group (Acute Control, Chronic Control and Chronic Keto) were investigated via multi-variate ANOVAs (dependent variables = 6 genes, fixed factor = diet grouping; Fig. 2) for mitochondrial related gene expression within each tissue type.

Diet group significantly impacted gene expression for *Cox5b*, a complex II associated gene, within the liver (F_(2,15)_ = 14.29, p = 0.001). Post-hoc analyses indicate *Cox5b* expression within the liver is significantly elevated in both Chronic Control (p < 0.001) and Chronic Keto (p = 0.003) relative to Acute Control (Fig. 2A). Another complex II associated gene, *Cox2*, was significantly altered by diet group within CA3 (F_(2,17)_ = 10.23, p = 0.002). Within CA3, post-hoc analyses indicate a specific effect of Chronic Keto increasing *Cox2* gene expression relative to both Acute Control (p = 0.002) and Chronic Control (p = 0.01), though Chronic Control and Acute Control did not differ (p = 0.75), indicating keto increases gene expression independent of TRF duration (Fig. 2B). Also within CA3, there was a significant effect of diet group on *Pgc1a* expression (F_(2,17)_ = 6.88, p = 0.008), a marker of mitochondrial biogenesis. *Pgc1a* gene expression in this region was elevated in Chronic Control relative to both Acute Control (p = 0.02) and Chronic Keto (0.01), but did not differ between Acute Control and Chronic Keto (p = 0.89), indicating potential increases in mitochondrial biogenesis provided by TRF are blocked by the keto diets. Gene expression did not differ across groups in any other tissue for any gene assessed (Fig. 2C–E).

## 4. Discussion

Herein we report that mitochondrial kinetics and mitochondrial-related gene expression analyses from brain, liver and muscle tissues taken from aged (~24 months) male rats fed an acute (~3 months) control time restricted feeding (TRF) diet (Acute Control), a chronic (~1.5 years) control TRF diet (Chronic Control) or chronic ketogenic Diet (keto) TRF diet (Chronic Keto) are differentially impacted in a tissue specific manner. Both keto diets and TRF converge on several overlapping features and pathways, including potential generation of ketone bodies and influence on SIRT1 [27–29]. While the study design herein was not designed to completely disentangle the two, chronic vs acute effects of TRF can be considered between the two control groups, whereas the effect of macronutrient ratio can be compared across the Chronic Control and Chronic Keto groups. While many effects of keto diets and chronic TRF presented herein resulted in alterations in the same direction relative to Acute Control TRF, there were some obvious trends driven by a single experimental group. Intriguingly, despite well documented benefits of TRF, a modestly acute (~3 months) bout of TRF while already in older age (~21 months) resulted in differential mitochondrial activity and related gene expression relative to chronic TRF administration. Interestingly, while gene expression within several subregions of the hippocampus (HPC) were quantified, effects were only observed within CA3 (and not CA1 or dentate gyrus).

Mitochondrial complex II, also known as succinate dehydrogenase (SDH), is a crucial component of the electron transport chain (ETC) in the mitochondria, playing a role in cellular respiration and energy production. Interestingly, we saw divergent effects on complex II activity across in the peripheral tissues studied investigated herein, with a significantly higher activity within the liver of Chronic Keto-fed rats and a strong trend towards less activity within the muscle of all Chronic TRF -fed rats relative to Acute Control fed rats. No significant alterations in complex II activity were observed within the HPC. As the liver is the primary site of ketogenesis, it is unsurprising that complex II activity was heavily impacted by Chronic Keto within this tissue. In fact, keto-induced increases in mitochondrial turnover are specific to hepatocytes and not observed within brain tissue of the same subjects [30].

Neither dietary intervention impacted gene expression of Succinate dehydrogenase complex flavoprotein subunit A (*Sdha*), which encodes a major catalytic subunit of complex II/SDH, within any tissue. While in a state of nutritional ketosis, the liver is the primary site for ketone body production, requiring additional metabolic resources. The relative elevation in activity within the liver of Chronic Keto-fed rats suggests enhanced energy production, as this complex contributes to the production of adenosine triphosphate (ATP), thereby providing more energy for cellular processes within the liver. Thus, an increase in complex II activity may indicate an adaptive response to the higher energy demands, reflecting an attempt to maintain mitochondrial function and efficiency in response to cellular needs or external stimuli.

Conversely, the trend towards less complex II activity within the muscle may be indicative of the use of an alternate fuel source by this tissue. While the liver cannot utilize ketone bodies because this tissue lacks the necessary enzyme beta ketoacyl-CoA transferase [31], the muscle readily utilizes ketone bodies for TCA cycle function [32]. These data align with previous reports of no change in muscular Complex II activity with keto diets [33], though data on the influence of keto on mitochondrial bioenergetics remains largely mixed [34–36]. *Sdha* plays a key role in the oxidation of succinate to fumarate, which is tightly linked with glucose metabolism, within the TCA cycle. Thus, it’s possible that decreased *Sdha* reflects metabolic adaptation away from glucose utilization towards ketone metabolism.

Regardless of dietary macronutrient composition, we observed significantly less complex IV activity within HPC and muscle tissues in Chronic TRF-fed rats relative to Acute Control. Complex IV, also known as cytochrome *c* oxidase (COX), is a key enzyme in the electron transport chain responsible for the final step of oxidative phosphorylation, playing a critical role in the transfer of electrons and the reduction of oxygen to water. Therefore, decreasing complex IV activity may be indicative of a reduced capacity to generate ATP. However, it’s important to consider whether the decrease in complex IV activity is a result of adaptive changes in response to the TRF regimen or if it reflects a compromised state. Some studies suggest that certain dietary interventions, including time-restricted feeding, can induce adaptive changes in cellular metabolism [37].

The expression of several genes related to mitochondrial complex IV were also quantified. Within the brain there was significantly higher *Cox2* gene expression carried mainly by Chronic Keto-fed rats relative to Acute Control. While the role of *Cox2* in neurodegenerative disease is debated [38], expression is necessary for proper learning and memory [39]. Expression of *Cox2* may also play a role in neuroprotection, though the time course of expression may influence whether this would be pro- or anti-inflammatory [40]. Moreover, our data are in contrast to previous reports of keto diet-induced decreases in *Cox2* gene expression in both human and animal models in multiple tissue types [41,42]. However, there was a notable trend for decreased expression within the liver by Chronic TRF relative to Acute TRF, in line with previous literature. Also within the liver was significantly higher *Cox5b* gene expression in both Chronic Control and Chronic Keto-red groups relative to Acute Control. *Cox5b* (Cytochrome *c* oxidase subunit 5B) is another component of the cytochrome *c* oxidase complex (Complex IV). This elevation may be indicative of the adaptation of the liver to more efficient energy production through the shift away from glycolysis towards fatty acid oxidation.

Both keto diets [43] and TRF [44] influence mitochondrial biogenesis. Therefore, we also quantified expression of two mitochondrial biogenesis-related genes, PCG1α and TFAM. While there were no differences in TFAM expression within any tissues, PCG1α was affected within the brain. Within the CA3 subregion of the HPC, PCG1α expression was higher in rats fed the Chronic Control diet, but not Chronic Keto, relative to Acute Controls, indicating that the keto diet inhibited the Chronic TRF-induced increase in expression. PCG1α is a master regulator of mitochondrial biogenesis, so these data could indicate more mitochondria in the HPC of Chronic Control rats, potentially leading to improved oxidative metabolism. Moreover, upregulation of this is neuroprotective against oxidative stress and cell damage and associated with neuronal maintenance [45,46].

Our data are in mixed agreement with the abundance of studies investigating these types of dietary interventions on mitochondrial function. While many, but not all, of these previously published reports differ on the direction of changes observed herein, it is important to note methodological differences in study design. To our knowledge, no other published studies have investigated the influence of keto diets with or without TRF on mitochondrial function in aged rats fed this diet for such a long duration. However, transcriptomics analysis of aging mice demonstrated how impactful 1 vs 14 months of dietary intervention are in the aged mouse brain [47], validating the importance of this line of work. Similarly, 32+% caloric restriction was insufficient to extend the median lifespan or prevent age-related lesion burden of rats when administered at 18 or 26 months of age onward [48,49]. Thus, our results likely reflect long term impacts of dietary intervention in the context of advanced age, unlike the vast majority of reports utilizing young, healthy subjects on a shorter-term diet. Sedentary rats utilized as control subjects in laboratory conditions overconsume, leading to an obese phenotype [50] as observed herein. Thus, it is possible that late life interventions are incapable of reversing the long-term impacts of overconsumption and obesity, despite weight loss in old age.

Our study was only able to include male rats, thus sex specific responses to these diets would likely provide different findings [51,52]. Moreover, while aged rats can get into a state of nutritional ketosis, and it is largely beneficial for their metabolic health [53], there is strong evidence that aged rats are impaired at metabolic switching and have different responses to keto diets/TRF [13,25,54,55]. Increased attention to peripheral mitochondrial dysfunction has revealed associations with many age-related diseases, including Alzheimer’s disease [7], strengthening the need for this type of multi-tissue investigation in aged subjects. In conclusion, our findings highlight the complex, tissue-specific responses to long-term dietary interventions in aged rats, emphasizing the need for further research to unravel the intricate mechanisms underlying these effects. These insights could pave the way for developing targeted nutritional strategies to enhance mitochondrial function and overall metabolic health in aging populations.

## Figures and Tables

**Table 1:** Group sizes utilized across mitochondrial complex (kinetic) assays and PCR assays.

		Liver	Muscle	HPC
**Acute Control**	Kinetic Assays	4	8	3
	PCR Assays	6	7	7
**Chronic Control**	Kinetic Assays	3	5	4
	PCR Assays	5	5	5
**Chronic Keto**	Kinetic Assays	3	6	4
	PCR Assays	5	6	6

**Table 2:** Gene sequences used to generate primers utilized herein.

Gene name (Gene symbol)	Descriptor	Forward Sequence	Reverse Sequence
Succinate dehydrogenase complex flavoprotein subunit A (S*dha*)	Complex II subunit	CCTCCGTGGTTGAGCTAGAA	CCTTTCCCGAACTTGAGGCT
Cytochrome *c* oxidase subunit 1 (*Cox1*)	Complex IV subunit	CTGTACTACGCCTGAGTTTCTG	CTTGAAGTGGGTCAGGATGTAG
Cytochrome *c* oxidase subunit 2 (*Cox2*)	Complex IV subunit	ACGTGTTGACGTCCAGATCA	GGCCCTGGTGTAGTAGGAGA
Cytochrome *c* oxidase subunit 5b (C*ox5b*)	Complex IV subunit	GAGATGGCTTCAAGGTTACTTCG	AGTAGGGACACCACCTCCAGA
Actin beta (*Actb*)	Housekeeping gene for normalization	ACAACCTTCTTGCAGCTCCTC	TCATCCATGGCGAACTGGTGG
Peroxisome proliferator-activated receptor gamma coactivator 1-alpha (P*gc1a*)	Mitochondrial biogenesis marker (“master regulator”)	TTGACTGGCGTCATTCAGGA	ACCAGGGCAGCACACTCTAT
Mitochondrial transcription factor (*Tfam*)	Mitochondrial biogenesis marker	CCAAAAAGACCTCGGTCAGC	AGCTTCAATTTCCCCTGAGC
